# Milk and infant plasma hormone concentrations predict sweet and fat taste acceptance and later food intake

**DOI:** 10.1111/jne.70282

**Published:** 2026-10-01

**Authors:** Ana Luz Kruger, Agustina Malpeli, Marisa Sala, Carla Casado, Ignacio Mendez, Lucrecia Fotia, Mercedes López, María Silvina Faquini, Daniel Castrogiovanni, Helgi Schioth, Sophie Nicklaus, Mario Perello, María F. Andreoli

**Affiliations:** ^1^ Instituto de Desarrollo e Investigaciones Pediátricas (IDIP). Children's Hospital HIAEP “Sor María Ludovica” La Plata ‐ Comisión de Investigaciones Científicas de la Provincia de Buenos Aires (CIC‐PBA) La Plata Buenos Aires Argentina; ^2^ CONICET La Plata Buenos Aires Argentina; ^3^ Laboratorio Central Children's Hospital HIAEP “Sor María Ludovica” La Plata La Plata Buenos Aires Argentina; ^4^ Neurophysiology Group Instituto Multidisciplinario de Biología Celular (IMBICE) (UNLP ‐ CIC‐PBA ‐ Consejo Nacional de Investigaciones Científicas y Técnicas (CONICET)) La Plata Argentina; ^5^ Department of Surgical Sciences, Functional Pharmacology and Neuroscience Uppsala University Uppsala Sweden; ^6^ UMR CSGA Université Bourgogne Europe, Institut Agro, CNRS, INRAE Dijon France

**Keywords:** infant hormones, LEAP2, milk hormones, taste acceptance

## Abstract

Early‐life exposure to metabolic hormones may influence the development of taste acceptance and subsequent intake of hyperpalatable foods (HPF) in infants, but evidence integrating hormonal, behavioral, and dietary data in humans is limited. Therefore, our objective was to determine whether concentrations of insulin, leptin, cortisol, and liver‐expressed antimicrobial peptide 2 (LEAP2) in human milk and infant plasma at 6 months predict infant sweet and fat taste acceptance and HPF intake later in infancy. In this prospective cohort study, human milk and infant plasma were collected at 6 months and analysed for insulin, leptin, cortisol, and LEAP2. At 18 months, sweet and fat taste acceptance were assessed using ingestion ratio (IR; motivational drive) and liking ratio (LR; hedonic response). HPF intake was quantified from standardized 24‐h dietary recalls. Associations were evaluated using multivariable linear regression. Eighty two mother–infant pairs were enrolled, of which 28 completed follow‐up and were included in the final analysis. Milk LEAP2 was positively associated with fat IR (*p* = .037). Milk insulin was inversely associated with sweet IR (*p* = .003) and LR (*p* = .020), while milk leptin was positively associated with sweet IR (*p* = .013) and LR (*p* = .018) and inversely associated with HPF intake consumption (*p* = .001). Milk cortisol was positively associated with fat (*p* = .043) and sweet IR (*p* = .014). In contrast, infant plasma LEAP2 was inversely associated with fat IR (*p* = .018) and HPF intake (*p* = .002) but positively associated with sweet IR (*p* = .005) and LR (*p* = .023). Infant plasma cortisol was directly associated with HPF intake (*p* = .006) and inversely associated with sweet and IR (*p* = .004) and fat LR (*p* = .009). Milk‐derived and endogenous metabolic hormones were differentially associated with taste acceptance and HPF intake, identifying specific hormonal pathways that may shape early feeding behavior and later food intake. Clinical Trial Registry: This study was registered at clinicaltrials.gov as NCT05798676. https://clinicaltrials.gov/study/NCT05798676.

## 1 INTRODUCTION

The first 2 years of life are a critical window for the establishment of taste preferences and dietary habits, which influence long‐term health and childhood obesity risk.^1^, ^2^ During this period, sensory experiences and hormonal cues contribute to programming appetite and feeding behavior.^3^ Like in adults, food intake during infancy is regulated by the integration of homeostatic and hedonic signals, which influence feeding patterns and modulate satiety mechanisms and the rewarding sensory properties of foods.^4^ Human milk is a dynamic bioactive fluid containing metabolic hormones that participate in early metabolic and behavioral programming.^5^, ^6^ While it is well established that flavor compounds derived from the maternal diet are transmitted through milk and shape infants' sensory acceptance,^3^ the contribution of milk‐borne hormonal signals to the modulation of infants' hedonic responses to energy‐dense tastes and subsequent food choices remains poorly understood.

Several hormones present in human milk—including insulin, leptin, cortisol, and liver‐expressed antimicrobial peptide 2 (LEAP2)—may constitute a key endocrine pathway for metabolic programming.^6^, ^7^ LEAP2, a novel antagonist ligand of the growth hormone secretagogue receptor (GHSR, or ghrelin receptor), has been shown to reduce food intake and glycemia,^8^, ^9^ and is also involved in reward and motivation.^10^, ^11^ Insulin and leptin signal satiety, potentially inhibiting the hedonic drive for palatable foods,^12^, ^13^ while cortisol, a stress hormone, is often linked to increased consumption of hyperpalatable foods (HPF) in adults.^14^ Together, these hormones play fundamental roles in the regulation of energy balance, satiety, and stress response. Beyond their established functions in adult metabolism, these hormones are also critical during lactation, influencing maternal adaptations and potentially shaping infant development. Insulin and adipokines are among the most extensively studied milk bioactive peptides for their associations with infant body weight and growth.^15^ Research on milk cortisol has primarily focused on its effects on infant behavior, temperament, and adiposity.^7^, ^16^, ^17^ More recently, we reported the presence of LEAP2 in human milk and its association with infant adiposity, suggesting a possible role in the regulation of food intake and energy storage.^6^ However, studies addressing the importance of milk hormones in later feeding outcomes are lacking. Milk‐borne hormones are known to act in taste bud cells,^18^, ^19^, ^20^, ^21^ as well as survive digestion and be absorbed in the infant gut,^5^ potentially complementing endogenous hormonal functions. Despite their regulatory roles in adult energy homeostasis being well established, evidence regarding their functions in infancy remains scarce. Whether these early hormonal signals influence infants' feeding behavior and food intake is a question that remains largely unanswered.

Understanding the mechanisms that link hormone levels and feeding behavior is particularly relevant in the context of increasing exposure to HPF—highly processed products combining fat, sugar, and/or salt in specific proportions that maximize reward.^22^ Excessive consumption of HPF during early childhood has been linked to poorer diet quality and increased risk of overweight and obesity.^23^ Early hedonic responses to sweet and fat tastes are key determinants of later food preferences and dietary patterns.^1^, ^2^, ^3^ Therefore, elucidating how hormonal concentrations in human milk and infant circulation relate to taste acceptance and HPF intake may help identify novel mechanisms by which breastfeeding influences long‐term eating behavior and body weight regulation.

In this prospective cohort study, we aimed to fill this gap by jointly examining the relationship between hormonal profiles in human milk and infant plasma at 6 months of age and food intake at 18 months. Specifically, we assessed how milk‐ and infant‐derived concentrations of insulin, cortisol, leptin, and LEAP2 predict taste acceptance (evaluated through ingestion ratio [IR], reflecting motivational drive, and liking ratio [LR], reflecting hedonic pleasure) and HPF consumption (assessed under natural dietary conditions). Furthermore, we explored whether associations between milk hormones and taste acceptance are mediated by corresponding hormone concentrations in infant plasma, thereby testing the hypothesis that these hormone signals from exogenous milk influence feeding behavior, either directly or indirectly through endogenous hormonal regulation. The primary objectives of this study were to determine the associations between insulin, cortisol, leptin, and LEAP2 concentrations in human milk and infant plasma at 6‐months postpartum and infants' sweet and fat taste acceptance or HPF consumption at 18 months; and to assess whether infant plasma hormone concentrations mediate the associations between milk hormone levels and taste acceptance outcomes. This study integrates biochemical, behavioral, and dietary data, providing novel insight into how early exposure to metabolic hormones through breast milk may shape taste acceptance development and food intake during infancy.

## 2 METHODS

### 2.1 Study design

For this prospective cohort observational study, lactating women were recruited at the “Mother and Healthy Children Outpatient Service”, Instituto de Desarrollo e Investigaciones Pediátricas (IDIP), HIAEP “Sor María Ludovica”, La Plata, Argentina. Two visits were coordinated, one at the six‐month health check of their infants and the other at 18 months of age. The first visit included anthropometric measurements, data collection from the maternal clinical record, milk sampling three hours after the last breastfeeding, and infant blood sampling after a 4‐h fast for assessment of insulin, cortisol, leptin, and LEAP2. At the second visit, infants were required to attend after a four‐hour fasting period and underwent a sweet and fat taste acceptance test, a 24‐h dietary recall, and anthropometric measurements. Ethical approval and informed consent were obtained. Trial registration: clinicaltrials.gov, NCT05798676. Further details in Supporting Information.

### 2.2 Sweet and fat taste acceptance test

#### 2.2.1 Tastant solutions

As in previous studies, a lactose solution was used for sweet taste acceptance and a fat emulsion for fat taste acceptance.^24^, ^25^ Each solution was prepared using mineral water (Saint Michel, La Plata, Argentina). The lactose molecule was selected for analysis because it is one of the primary molecules responsible for the sweet taste of human milk. The 0.2 M lactose concentration was utilized, corresponding to its natural concentration in human milk.^26^ A mixture of equal parts of sunflower oil (Carrefour, Argentina) and rapeseed oil (Carrefour, Argentina) in mineral water at a concentration of 3.5% (w/v) was prepared as a fat emulsion. To facilitate homogenization of the emulsion, soy lecithin (MacroSalud, Argentina) was added at a concentration of 1% (w/v). The stability of the fat emulsion was ensured by blending the emulsion with a handheld mixer (BF‐HB300C BKF, Argentina) for 20 min. The solutions were prepared the day before the sessions and stored at 4°C.

#### 2.2.2 Procedure

The method was adapted from previous studies from our group.^24^, ^25^, ^27^ The test comprised a single session per taste. The fat emulsion was always evaluated after the sweet taste session, because of the potential coating phenomenon in the mouth.^24^ The experimenter presented the infant with four bottles—water, followed by a sweet tastant solution, then a second bottle of the same sweet tastant solution, and finally water. Each bottle was offered individually over a 45‐s period, with a 15‐s interval between presentations. The same procedure was repeated using the fat tastant solution. For each bottle, intake was quantified by weighing bottles before and after the test. The experimenter, who was blind to the tastant, evaluated the infant's liking through the infant's global behavior by using a 5‐point scale with the following specifications: 1 = “strong rejection”; 2 = “slight rejection”; 3 = “neutral reaction”; 4 = “slight acceptance”; and 5 = “strong acceptance”.^25^ The instruction for the experimenters was to consider the infant's global behavior, including his/her facial expressions. For each tastant, an IR, based on the consumed volumes, and a LR, based on the experimenters' judgment of the infant's liking, were calculated and represented the acceptance of the tastant relative to water. Both ratios range from 0 to 1, where values >0.5 indicate preference, <0.5 indicate rejection, and 0.5 indicates indifference. Further details in Supporting Information.

### 2.3 Twenty‐four‐hour dietary recall and classification of hyperpalatable foods

A 24‐h dietary recall of the infant's intake was conducted.^28^ Based on the HPF classification criteria proposed by Fazzino et al.,^22^ the foods consumed were categorized into: fat and sodium combination (F‐SOD); carbohydrates and sodium combination (C‐SOD); and fat and simple sugars combination (FS), and the percentage of items in each category was calculated. Further details in Supporting Information.

### 2.4 Statistical methods

Analyses were performed in R (v4.3.0). Data were summarized using appropriate descriptive statistics. Multiple linear regression models examined associations between milk/infant hormones and IRs, LRs, or HPF categories, adjusting for predefined confounders. Effect sizes were expressed as Cohen's *f* ^2^. Mediation analyses evaluated whether infant plasma hormones mediated associations between milk hormones and taste acceptance, using bootstrap confidence intervals (CIs)—(Figure S1). A post‐hoc directed acyclic graph (DAG) was produced to illustrate significant relationships. Significance was set at *p* < .05. Further details in Supporting Information.

## 3 RESULTS

### 3.1 Cohort characteristics

A total of 725 mother–infant pairs volunteered and were assessed for eligibility. Of these, 82 pairs were included in the cohort at 6 months postpartum, and 51 of these pairs completed the follow‐up at 18 months. Due to factors such as infant refusal to drink from a bottle, inability to complete the test sequence, or not meeting intake criteria, the final number of infants included who completed the taste acceptance test was 28 (Figure S2). Maternal characteristics, milk and infant hormone concentrations, anthropometric measures, and behavioral assessments are presented in Table 1. The infants included in the final analysis had similar characteristics to the total number of infants initially enrolled in the study (data not shown). Mothers had a mean pre‐pregnancy body mass index (BMI) of 24.9 kg/m^2^, 26% were primiparous, and 33.3% of the infants were females. In the taste acceptance test, sweet IR values were below 0.5, whereas sweet LR values exceeded 0.5, but no statistical differences were observed. Both fat IR and fat LR values remained below 0.5. Regarding food items consumed by infants, 28.4% were HPF, comprising 5.1% F‐SOD, 9.8% C‐SOD, and 10.3% FS. Examples of commonly consumed HPF: powdered or fluid milk, bread, yogurt, sweet cookies, plain crackers, cream cheese, and grated cheese. The associations between hormone concentrations in milk and infant plasma were also tested. No significant relationship was observed for insulin [β (95% CI): 0.05 (−0.55, 0.66), *p* = .848], and leptin [β (95% CI): 0.53 (−0.14, 1.19), *p* = .111]; a positive association was observed for cortisol [β (95% CI): 0.50 (0.10, 0.91), *p* = .019]; and a negative relationship for LEAP2 [β (95% CI): −0.38 (−0.70, −0.05), *p* = .029] after adjustments.

**TABLE 1:** Descriptive characteristics of the study cohort, ingestion ratio (IR) and liking ratio (LR) for sweet and fat taste and hyperpalatable foods consumption.

	Mean/Median/Frequency	Standard deviation/Q1–Q3/%
Maternal characteristics	*n* = 28	
Pre‐pregnancy BMI (kg/m^2^)^1^	24.92	4.17
Parity, primipara^2^	7	26%
IOM GWG guidelines ^2^		
Below or within (adequate)	19	70.37%
Exceed	8	29.63%
At 6 months postpartum	*n* = 28	
Age (years)^1^	27.88	5.32
BMI^1^	25.89	4.66
Milk insulin (μU/ml) ^3^	2.96	2.19, 5.27
Milk cortisol (ug/dl) ^3^	2.30	1.21, 2.95
Milk leptin (pg/ml) ^3^	0.19	0.11, 0.29
Milk LEAP2 (ng/ml) ^1^	2.00	0.69
Infant characteristics	*n* = 28	
At 6 months		
Sex (female)	9	33.33%
Weight (g) ^1^	7933	849
Length (cm) ^1^	66.92	1.57
BMI *z*‐score ^1^	0.28	1.04
Length *z*‐score ^1^	−0.19	0.81
Weight *z*‐score ^1^	0.11	1.01
Plasma insulin (μU/ml) ^3^	1.90	1.00, 2.55
Plasma cortisol (ug/dl) ^1^	11.34	5.62
Plasma leptin (ng/ml) ^3^	1.92	1.22, 2.50
Plasma LEAP2 (ng/ml) ^1^	8.00	2.24
At 18 months	*n = 28*	
Weight (g) ^1^	10,815	1132
Length (cm) ^1^	81.17	2.25
BMI *z*‐score ^1^	0.27	0.90
Length *z*‐score ^1^	−0.35	0.85
Weight *z*‐score ^1^	−0.02	0.88
Taste acceptance test		
Sweet taste		
Ingestion ratio (IR) ^1^	0.42	(0.23)
Liking ratio (LR) ^3^	0.53	(0.44, 0.58)
Fat taste		
Ingestion ratio (IR) ^3^	0.30	(0.19, 0.41)
Liking ratio (LR) ^1^	0.30	(0.16)
Hyperpalatable Foods Consumption (%)		
% Total HPF ^1^	28.41	11.01
% F‐SOD^3^	5.13	(0.00, 7.85)
% C‐SOD^1^	9.77	5.97
% FS^3^	10.26	(7.14, 15.71)

*Note*: Data presented as mean (SD) (1), frequency (%) (2), or median (Q1, Q3) (3).

Abbreviations: BMI: body mass index; BW/GA z‐score: birthweight for gestational age *z*‐score; C‐SOD: carbohydrates and sodium; FS: fat and simple sugars; F‐SOD: fat and sodium; HPF: hyper‐palatable foods; IOM GWG guidelines: Institute of Medicine guidelines for gestational weight gain adequacy; SD, standard deviation.

### 3.2 Associations of sweet and fat taste acceptance with milk hormone concentrations

No associations were found between either sweet IR or LR and milk LEAP2 concentration (Figure 1A and Table S1). Sweet IR and LR values were inversely associated with milk insulin concentration and positively associated with milk leptin concentration after adjustments. Sweet IR was also positively associated with milk cortisol concentration after adjustment. Fat IR was positively associated with milk LEAP2, insulin, and cortisol concentrations, while LR was inversely associated with milk insulin concentration. No associations were found between either fat IR or LR and milk leptin concentration.

**FIGURE 1:**
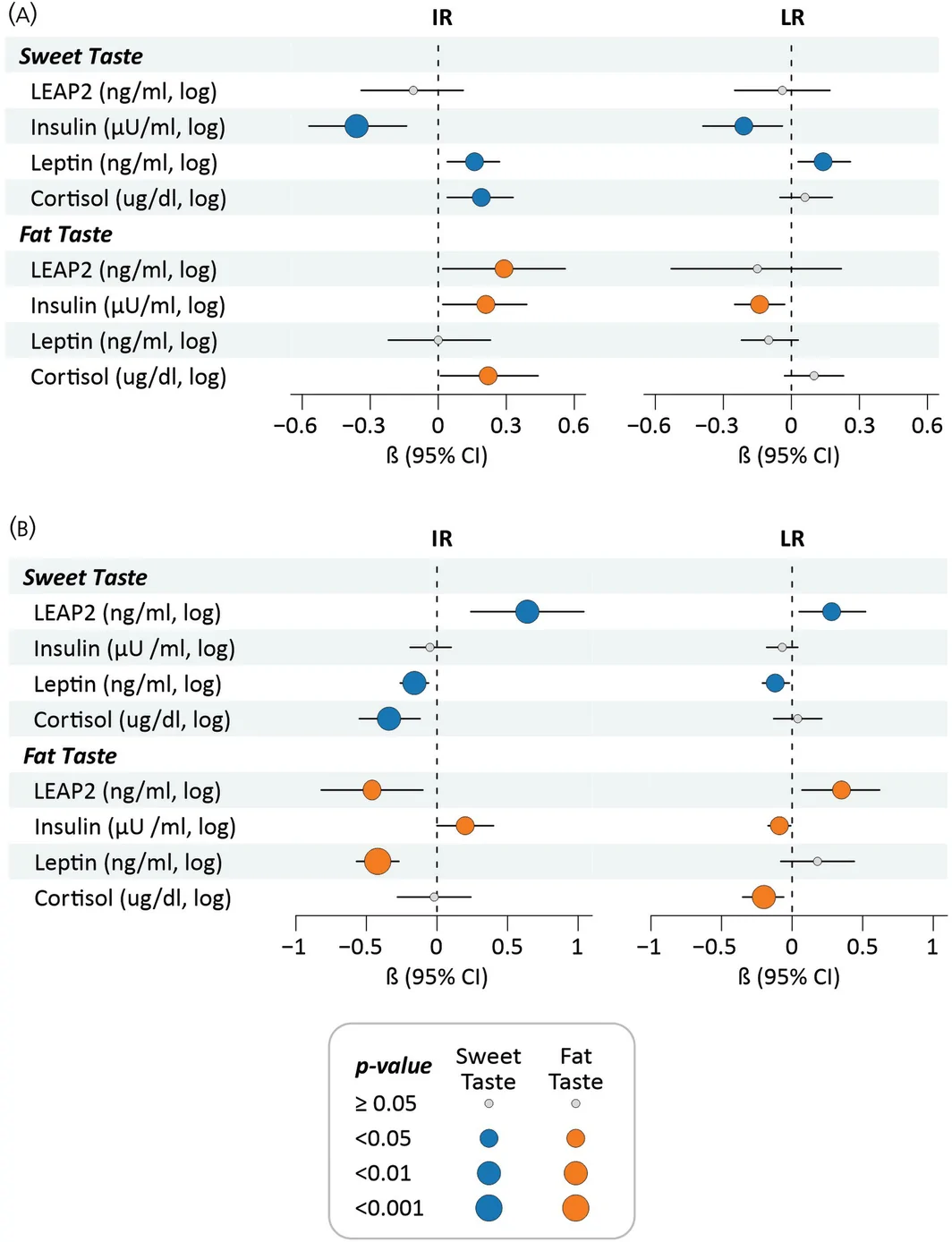
Forest plot showing the estimated regression coefficient β (95% CI) from linear regression analyses for associations between milk (A) and infant plasma (B) hormone concentrations as predictors of sweet and fat taste IR and LR. CI, confidence interval; IR, intake ratio; LR, liking ratio.

### 3.3 Associations of sweet and fat taste acceptance with infant plasma hormone concentrations and anthropometric measurements

Both sweet IR and LR were positively related to infant plasma LEAP2 concentration and inversely associated with infant plasma leptin concentration after adjustments (Figure 1B and Table S2). Sweet IR and LR values were inversely associated with infant plasma insulin concentration; however, these associations did not remain significant after adjustment. Sweet IR remained inversely associated with infant plasma cortisol concentration. A mediation analysis was performed to examine whether the association between milk cortisol concentration and sweet IR (Table S1) was mediated by infant plasma cortisol concentration. No statistically significant mediation (*p* = .180) or direct effect (*p* = .570) was found (Figure 2A).

**FIGURE 2:**
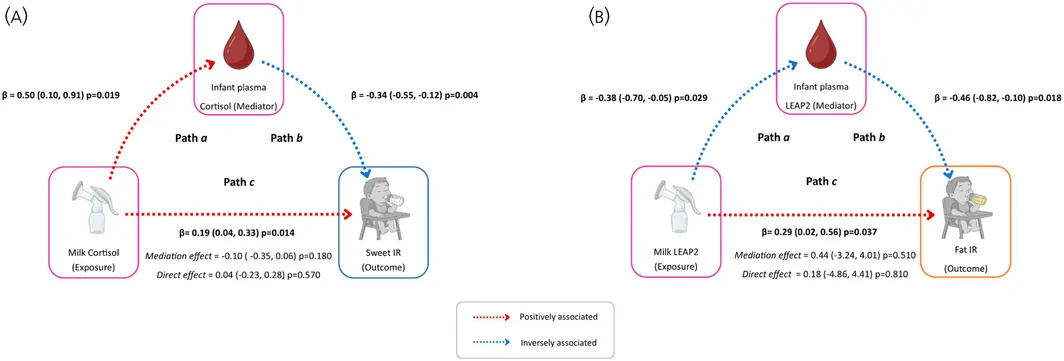
Mediation models assessing whether infant plasma hormone concentrations mediate associations between milk hormones and infant taste acceptance. Illustrations denote the exposure (milk), mediator (infant plasma), and outcomes (sweet and fat taste IR). (A) Infant plasma cortisol does not mediate the association between milk cortisol concentrations and sweet taste IR. (B) Infant plasma LEAP2 does not mediate the association between milk LEAP2 concentrations and fat taste IR. Arrows depict hypothesized paths: *Path a* (exposure → mediator), *path b* (mediator → outcome), and *path c* (total exposure → outcome). Regression coefficients β (95% CI) and *p*‐values are displayed for each path. Mediation and direct effects were estimated using bias‐corrected bootstrapped CIs. Statistically significant values appear in bold. CI, confidence interval; LEAP2, liver‐expressed antimicrobial peptide 2; IR, intake ratio.

Fat IR was inversely associated with LEAP2 and leptin concentrations, while fat LR was positively related with LEAP2 concentration after adjustments (Table S2). Fat IR was positively associated with infant plasma insulin concentration, while fat LR was inversely related with insulin and cortisol concentrations. A mediation analysis was conducted to determine whether the association between milk LEAP2 concentration and fat IR (Figure 1A and Table S1) was mediated by infant plasma LEAP2 concentration, and no statistically significant mediation (*p* = .510) or direct effect (*p* = .810) was observed (Figure 2B), indicating that infant plasma LEAP2 concentration did not mediate this relationship. No associations were observed between fat LR and IR and BMI *z*‐score, and sweet LR was inversely associated with BMI *z*‐score (Table S2).

### 3.4 Associations of HPF intake with milk hormone concentrations

Total HPF intake was inversely associated with milk leptin concentration, while F‐SOD intake was inversely associated with milk insulin concentration after adjustments (Figure 3, Table S3). In contrast, C‐SOD and FS intake were positively associated with milk LEAP2 concentration. No associations were found between total HPF, F‐SOD, C‐SOD, and FS intakes with milk cortisol concentrations.

**FIGURE 3:**
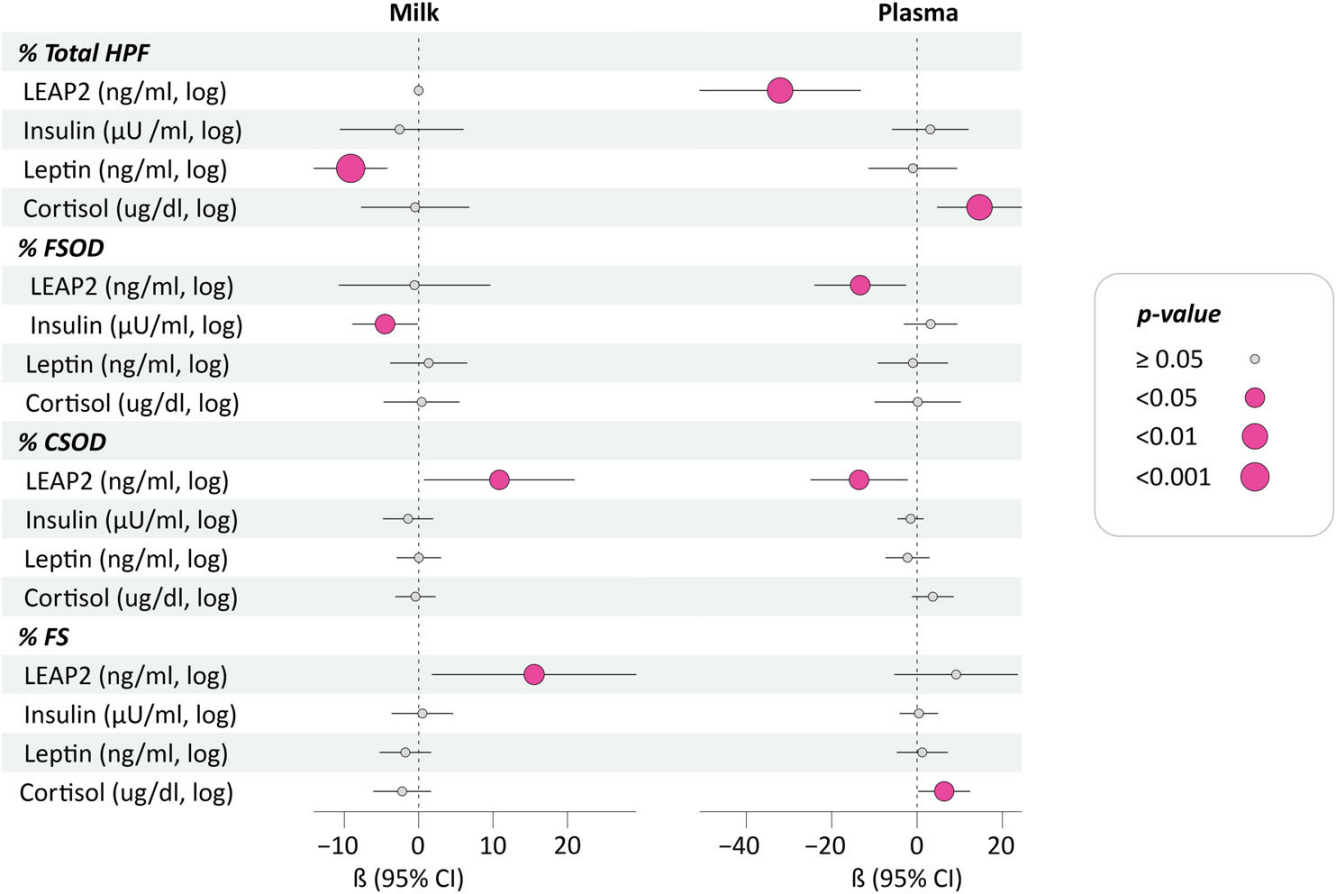
Forest plot showing the estimated regression coefficient β (95% CI) from linear regression analyses for associations between milk and infant plasma hormone concentrations as predictors of total HPF, F‐SOD, C‐SOD, and FS intake. CI, confidence interval; C‐SOD: carbohydrates and sodium combination; FS: fat and simple sugars combination; F‐SOD: fat and sodium combination; HPF: hyperpalatable food.

### 3.5 Associations of HPF intake with infant hormone concentrations and anthropometric measurements

HPF intake was inversely related to infant plasma LEAP2 concentration and was positively associated with infant plasma cortisol concentrations after adjustments (Figure 3, Table S4). F‐SOD and C‐SOD intakes were inversely associated with infant plasma LEAP2 concentration after adjustments. FS intake was positively associated with infant plasma cortisol concentration. No associations were found between total HPF, F‐SOD, C‐SOD, and FS intakes and infant plasma insulin or leptin concentrations. No associations were observed between HPF intake and BMI *z*‐score (Table S4).

## 4 DISCUSSION

To our knowledge, this is the first study integrating biochemical, behavioral, and dietary data to address how specific metabolic hormones during breastfeeding shape taste acceptance development and food intake during early infancy. We found that hormonal signals present in both human milk and infant plasma at 6 months were associated with taste acceptance patterns and HPF intake at 18 months (Figure 4). Notably, exogenous hormone signals delivered through milk showed different associations from those of infants' endogenous circulating hormone levels. Our results highlight the important role of milk‐derived hormones in shaping early taste acceptance, expanding current evidence that has largely focused on the influence of dietary flavor compounds transmitted through human milk.^29^

**FIGURE 4:**
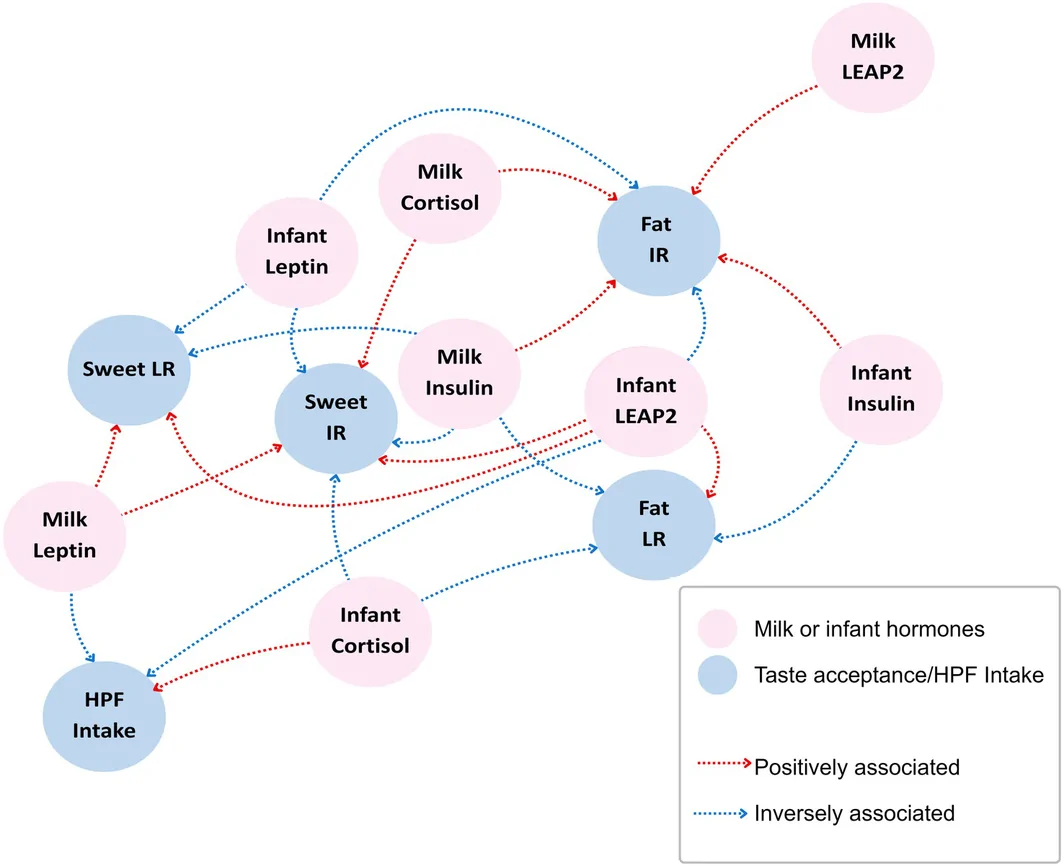
Post hoc directed acyclic graph (DAG) illustrating the significant associations observed between milk and infant plasma hormones and subsequent infant taste acceptance and HPF intake. Variables included in the final regression models (infant taste acceptance outcomes or HPF intake in blue and milk and infant plasma hormone concentrations in pink) are represented as nodes, with arrows indicating statistically significant associations. HPF, highly palatable foods; IR, intake ratio; LR, liking ratio.

Our findings suggest, for the first time, a potential role of early‐life LEAP2 levels in shaping taste acceptance and HPF intake. LEAP2 concentration in human milk was positively associated with fat IR, indicating a greater motivational drive toward fat consumption, which is consistent with the observed positive association with FS food intake, despite a lack of association with FSOD intake. Previous work from our group^6^ reports that milk LEAP2 is positively associated with adiposity in 6 month‐old infants. Taken together, these findings support the notion that milk‐derived LEAP2 may contribute to infant fat accretion by promoting intake of fat‐ and sugar‐rich foods. Infant plasma LEAP2 was inversely associated with fat IR and HPF intake (total and F‐SOD and C‐SOD categories, despite no association with FS). This pattern aligns with findings in adults, where circulating LEAP2 is inversely associated with energy intake^30^ and hunger,^31^ and positively associated with reward responsiveness,^30^ supporting its role in regulating energy balance and feeding behavior.^8^ Strikingly, however, infant plasma LEAP2 showed strong positive associations with sweet and fat LR, suggesting enhanced hedonic responsiveness to these tastes. The mechanisms mediating the distinct associations of LEAP2 present in milk or plasma with taste acceptance and later food intake require further investigation. Plasma LEAP2 likely reduces food intake and modulates reward‐driven feeding through its antagonistic action on GHSR, which is abundantly expressed in brain regions involved in reward and motivation.^8^, ^9^ GHSR expression has also been detected in taste bud cells, where it enhances fat taste responsiveness in mice,^18^ supporting the possibility that LEAP2 could modulate taste processing also through peripheral pathways. In rodents, central administration of LEAP2 reduces food intake, binge consumption of high‐fat diets,^10^ and preference for peanut butter.^11^ Taken together, these observations point to LEAP2 as a differential modulator of taste acceptance and feeding behavior, with potentially distinct effects depending on whether it is supplied exogenously via human milk or produced endogenously by the infant, acting through different pathways.

Milk insulin concentration was inversely associated with sweet taste acceptance (IR and LR), consistent with its proposed role in central satiety signaling and inhibition of sweet reward pathways.^12^, ^13^ Interestingly, milk insulin concentration also showed an inverse association with fat LR, but it was positively associated with fat IR. Infant plasma insulin also showed opposite associations with fat LR and IR. These findings suggest that insulin may differentially modulate macronutrient‐specific hedonic responses, potentially attenuating sweet reward sensitivity while enhancing the drive to consume fatty tastes under certain endocrine, metabolic, or developmental conditions. Although the molecular mechanisms through which insulin modulates taste acceptance remain unclear, evidence from animal models shows that insulin receptors are also expressed in taste cells.^19^ Moreover, circulating insulin may regulate taste cell growth and/or proliferation through activation of the mTOR pathway,^32^ suggesting a potential biological route by which insulin could influence taste function. Therefore, our results point to a selective influence of insulin on early sensory and motivational aspects of feeding behavior, rather than on overall energy‐dense food consumption.

Milk leptin was positively associated with sweet but not fat taste acceptance. Evidence linking milk leptin to taste acceptance is limited. While prior research has largely focused on the debated effects of milk leptin levels on infant body weight and adiposity,^15^ our novel findings indicate that milk leptin, as an exogenous source, may positively influence sweet intake, potentially contributing to its regulatory effect on infant body weight and adiposity.^15^ Milk leptin was inversely associated with HPF consumption, with this association reaching very strong statistical significance (*p* = .001), despite no association with HPF categories. This aligns with leptin's well established anorexigenic role, supporting the idea that its greater exposure through breast milk may reduce the intake of energy‐dense, highly palatable foods later in infancy. In contrast, higher infant plasma leptin concentration was associated with lower sweet (LR and IR) and fat (IR) acceptance, consistent with leptin's inhibitory effects on hedonic feeding responses and appetite regulation.^12^, ^13^ While evidence in infants is scarce, studies in adults with diabetes mellitus show that salivary leptin level correlates inversely with sweetness perception.^33^ Experimental data in rodents support a modulatory role of leptin in taste‐driven feeding: leptin suppresses behavioral responses to sweet tastants through activation of leptin receptors in taste cells,^20^, ^21^ and leptin receptor‐deficient mice show no suppression of sucrose responses following leptin antagonist administration.^34^ Evidence regarding leptin's role in fat sensing is limited; one study in mice reports that lingual leptin inhibits oro‐sensory detection of linoleic acid by interfering with calcium signaling and membrane potential in taste cells.^35^ These findings are consistent with evidence in rats showing that leptin intake during lactation influences later food preferences and protects against obesity development.^36^ Therefore, milk leptin may serve as an early endocrine signal that modulates sweet‐taste responses and shapes later susceptibility to energy‐dense food consumption, potentially linking early sensory experiences with long‐term metabolic outcomes.

Our findings show that milk cortisol was positively associated with IR for both sweet and fat tastants, suggesting that glucocorticoid exposure through milk may facilitate approach behaviors or exploratory feeding responses in infants. Glucocorticoid exposure via milk has been reported to shape stress reactivity, infant temperament, body weight, and adiposity,^7^, ^16^, ^17^ but, to our knowledge, this is the first study to report associations with infant feeding behavior. In contrast to the effects of milk cortisol, higher endogenous cortisol concentration was associated with lower sweet IR and fat LR, yet with higher HPF intake—particularly FS‐type foods, with no association with either F‐SOD and C‐SOD. The direct association with HPF intake is consistent with evidence that elevated circulating cortisol promotes consumption of high‐fat, high‐sugar foods as a stress‐coping mechanism through activation of reward‐related pathways.^37^, ^38^ Such effects, however, have been documented primarily in adults under stress, a context that differs substantially from early‐life physiology, and information about infants is lacking. Experimental disruption of the hypothalamic–pituitary–adrenal axis (e.g., adrenalectomy) reduces sweet preferences, and hormone replacement (e.g., dexamethasone, deoxycorticosterone acetate) restores them,^39^ underscoring the potent regulatory role of glucocorticoids in ingestive behavior. Therefore, milk‐derived and endogenous cortisol may influence infant feeding behavior through distinct pathways, highlighting the complexity of glucocorticoid signaling in early appetite regulation.

Interestingly, the hormonal patterns associated with HPF intake did not fully align with those observed for taste acceptance. Milk leptin was positively associated with sweet taste acceptance, but it showed an inverse relationship with HPF consumption. Similarly, infant plasma LEAP2 was positively associated with sweet and fat acceptance, contrasting with its negative association with HPF intake. In turn, infant plasma cortisol showed the opposite pattern, being inversely related to sweet and fat acceptance and directly associated with HPF intake. Moreover, neither milk nor infant insulin levels—despite their associations with taste acceptance—were related to HPF intake. These opposing patterns align with prior observations showing no strong associations between infants' exposure to sweet and fatty foods and their liking for these tastes later in childhood.^40^ They may also reflect distinct regulatory mechanisms of appetite and taste preference during early development, prior to the full establishment of reward‐driven feeding behaviors. Together, these findings underscore that taste acceptances represent only one of several factors that contribute to multifaceted feeding behaviors. The lack of concordance between taste acceptance and actual food intake highlights the complexity of translating early sensory responses into real‐world dietary behaviors. Taste tests capture immediate hedonic and motivational responses under controlled conditions, whereas HPF intake reflects more complex behaviors shaped by central cognitive processes, parental feeding practices, and food availability. Also, our results showed that taste acceptance measures and HPF intake were generally not associated with BMI *z*‐score at 18 months. While this may suggest that these behavioral measures do not directly translate into differences in body size at this early age, anthropometric outcomes are influenced by multiple biological and environmental factors and may therefore not adequately reflect taste preferences or short‐term dietary behaviors. Nevertheless, these measures remain valuable tools for assessing distinct dimensions of feeding behavior, including hedonic and motivational responses to taste and the potential influence of early‐life metabolic hormone exposure.

Despite the biological plausibility that infant hormone concentrations may mediate the effect of milk hormones on taste acceptance, our mediation analyses did not support this hypothesis. Previous results from our group^7^ show that milk cortisol mediates the association between maternal plasma and infant salivary cortisol concentrations. This effect does not seem to be extensive to behavioral observations, as neither infant plasma cortisol nor LEAP2 mediated the observed associations between their corresponding milk concentrations and taste acceptance. This suggests that the effects of milk hormones may involve other mechanisms, such as peripheral pathways involving receptor‐level signaling in taste bud cells that express the corresponding hormone receptors.^18^, ^19^, ^20^, ^21^ Another possibility is that milk hormones act indirectly through influencing infant body weight and adiposity^5^, ^6^, ^15^ or microbiota composition,^41^, ^42^ which were not directly assessed here. Also, the extent to which milk‐borne hormones influence their corresponding circulating concentrations in infants remains unclear and may differ according to the hormone considered. Although the mechanisms of milk components absorption in the infant gut have been studied,^5^ the dynamics and transitory alterations in infant plasma remain mostly unknown. Our results revealed a positive association between exogenous milk and endogenous infant plasma cortisol, whereas no significant associations were observed for leptin or insulin and an inverse association was found for LEAP2. These findings suggest that the relationship between milk‐borne and infant endogenous hormones is unlikely to be explained solely by acute transfer into the circulation, and may vary by hormone. Consequently, the pathways linking milk hormones to later taste acceptance may extend beyond changes in circulating hormone concentrations measured at a single time point.

A major strength of this study is the integration of hormonal, behavioral, and dietary data within the same cohort, allowing a comprehensive assessment of how early hormonal exposures relate to later feeding outcomes. Conducting in‐person infant taste assessments presents significant logistical and ethical challenges, underscoring the value of this dataset. The use of both IRs and LRs provides a nuanced measure of taste acceptance, and the study design enabled adjustment for key maternal and infant confounders. Nevertheless, several limitations exist. The modest sample size reduces statistical power and generalizability, and the observational design limits causal inference. However, a priori power calculations indicated sufficient power to detect the large effect sizes observed (Cohen's *f* ranging between 0.40 and 0.55 for the main associations). Dietary intake at 18 months was based on a single 24‐h recall, which may not reflect habitual intake. Additionally, plasma ghrelin concentration could not be measured, and hormone assessments at 6 months may not capture variation across lactation.

In summary, our findings provide novel evidence that hormonal signals in breast milk and infant plasma during early life are associated with later taste acceptance and consumption of HPF. The lack of mediation through infant plasma hormones suggests that milk‐derived endocrine signals may exert direct, independent effects on the developing appetite control system. The large effect sizes observed (Cohen's *f* >0.40) and the corresponding power estimates reinforce the reliability of the associations detected. Together, these results offer unique insight into how breastfeeding may program long‐term feeding behavior and food intake via specific hormonal pathways.

## AUTHOR CONTRIBUTIONS

**Ana Luz Kruger:** Conceptualization; methodology; data curation; writing – original draft; investigation. **Agustina Malpeli:** Investigation. **María Silvina Faquini:** Investigation. **Helgi Schioth:** Funding acquisition; investigation. **Mercedes López:** Investigation. **Marisa Sala:** Investigation. **Sophie Nicklaus:** Methodology; investigation; writing – review and editing. **Ignacio Mendez:** Investigation. **Carla Casado:** Investigation; data curation. **Daniel Castrogiovanni:** Investigation. **Mario Perello:** Conceptualization; investigation; writing – review and editing; funding acquisition. **Lucrecia Fotia:** Investigation. **María F. Andreoli:** Conceptualization; investigation; funding acquisition; writing – review and editing; writing – original draft; methodology.

## FUNDING INFORMATION

This work was supported by grants from Fondo para la Investigación Científica y Tecnológica, Argentina (FONCyT, PICT2019‐3054 and PICT2020‐3270 to MP, PICT2019‐2256 and PICT2021‐0121 to MFA), the Novo Nordisk Foundation (Nr. 0092558) and the Wenner Gren Stiftelserna (GFh2025‐0013 and GFh2025‐0021). The funders had no role in the design, implementation, analysis, and interpretation of the data.

## CONFLICT OF INTEREST STATEMENT

The authors declare no conflicts of interest.

## Supporting information


**Figure S1.** Conceptual diagram of the investigated mediation analyses.
**Figure S2.** Flowchart of participants' recruitment.
**Table S1.** Associations of infant sweet and fat taste acceptance (IR and LR) with milk insulin, cortisol, leptin, and LEAP2 concentrations.
**Table S2.** Associations of infant sweet and fat taste acceptance (IR and LR) with plasma hormone concentrations and anthropometric measurements.
**Table S3.** Associations of milk hormone concentration with hyperpalatable foods intake.
**Table S4.** Associations of HPF intake with infant hormone concentrations and anthropometric measurements.

## Data Availability

The data that support the findings of this study are available on request from the corresponding author. The data are not publicly available due to privacy or ethical restrictions.
