# Supplementary material for: Milk and infant plasma hormone concentrations predict sweet and fat taste acceptance and later food intake
Source: J Neuroendocrinol. 2026 Oct 1;38(10):e70282. doi: 10.1111/jne.70282 (PMC13631378; doi:10.1111/jne.70282)
Supplement: Supplementary file 1 [file JNE-38-e70282-s001.docx]

**Milk and infant plasma hormone concentrations predict sweet and fat taste acceptance and later food intake**

Ana Luz Kruger, Agustina Malpeli, Marisa Sala, Carla Casado, Ignacio Mendez, Lucrecia Fotia, Mercedes López, María Silvina Faquini, Daniel Castrogiovanni, Helgi Schioth, Sophie Nicklaus, Mario Perello, María F. Andreoli

**Supplemental Methods**

*2.1 Subjects and study design*. Data and samples were obtained from a larger clinical trial evaluating maternal characteristics associated with child growth and adiposity (1). Lactating women were recruited at the six-month health check of their infants at the ´Mother and Healthy Children Outpatient Service´, Instituto de Desarrollo e Investigaciones Pediátricas (IDIP), HIAEP “Sor María Ludovica”, La Plata, Argentina. Enrolment into the study occurred between May 2019 and February 2023. The inclusion criterion was full breastfeeding of the infants (defined as exclusive or predominant breastfeeding, not including formula feeding (2)) from birth to 6 months old, the end of exclusive breastfeeding, to ensure a high intake of milk hormones by the infants in the first half year of life. Exclusion criteria were formula feeding, diabetes (Type I or II or gestational), multiple pregnancy, hypertensive disorders during pregnancy, and delivery of a pre-term infant (<37 weeks). Two visits were coordinated, one at 6 months and the other at eighteen months of age of the infant. Women who agreed to participate arranged a first visit to the outpatient service at 6 months postpartum between 8:00 and 10:00 AM after an 8-hour fast. The first visit included anthropometric measurements, milk sampling, and blood sampling on the infant, as well as data collection from the maternal clinical record, including parity, weight in the first trimester, and weight in the week prior to delivery. First-trimester weight was used to determine the pre-pregnancy body mass index (BMI). Gestational weight gain (GWG) was calculated as the difference between maternal weight at delivery and maternal weight at the first trimester, and was dichotomized as “below or within” versus “above” the Institute of Medicine (IOM) 2009 guidelines (3). At the second visit, scheduled when the infants were 18 months old, infants underwent a sweet and fat taste acceptance test, a 24-hour dietary recall, and anthropometric measurements. Infants were required to attend after a four-hour fasting period. Only data and plasma samples from participants who completed the taste acceptance test were included in this study (n = 28). The study was approved by the Institutional Committee for the Revision of Research Protocols (CIRPI, IDIP) and conducted according to the Declaration of Helsinki guidelines and Argentinian legal provisions governing clinical research on humans. Written informed consent was obtained from all participants. This trial was registered at clinicaltrials.gov as NCT05798676.

2.2 *Sample collection*. At the first visit, women provided a single, complete breast milk expression sample three hours after the last breastfeeding using a sterile collection shield and container attached to an electric breast pump (NUK Luna Electric Breast Pump) as previously reported (1, 4). Skim milk was generated by spinning milk at 3,000g for 20 min and immediately analyzed for insulin, cortisol, leptin, and LEAP2 concentration. Infant blood samples were also drawn and collected in heparinized tubes for plasma separation and stored at -80°C until analysis.

*2.3 Biochemical analysis*. In infant plasma as well as in skim milk, insulin and cortisol were assessed using a chemiluminescence immunoassay (Access 2, Beckman Coulter, intra-assay CV 2.0–4.2% and inter-assay CV<6% for insulin and intra-assay CV 4.4–6.7% and inter-assay CV<8% for cortisol). Leptin levels were assessed using the commercially available kit Human Leptin ELISA DuoSet (R & D Systems, Minneapolis, MN, USA, intra-assay CV 5.1% and inter-assay CV 5.6%). LEAP2 was detected using a specific enzyme immunoassay from Phoenix Pharmaceutical (EK-075-40, intra- and inter-assay variations of 6.4 and 9.9 %, respectively), following the manufacturer’s instructions.

*2.4 Anthropometric measurements*. Maternal height and weight were measured in the first visit. BMI was calculated by dividing weight by height squared (kg/m^2^). Infant anthropometric data included body weight and length at 6 and 18 months of age. Length, weight, and BMI values were converted to sex- and age-specific standard deviation scores (z-scores) using WHO AnthroPlus software version 1.0.4 (World Health Organization). Standard measurement procedures and techniques were applied in all anthropometric measurements (5, 6).

*1. Taste acceptance test*

*Procedure*. The method was adapted from previous studies from our group (7-9). The test comprised a single session per taste. The fat emulsion was always evaluated after the sweet taste session, because of the potential coating phenomenon in the mouth (7). The experimenter presented the infant with four bottles—water, followed by a sweet tastant solution, then a second bottle of the same sweet tastant solution, and finally water. Each bottle was offered individually over a 45-second period, with a 15-second interval between presentations. The same procedure was repeated using the fat tastant solution. The experimenter, the mother, and the infant were seated at the same table. The infant was permitted to handle the bottle independently under the close supervision of the experimenter. To enhance compliance, the mother was requested to administer the bottle herself, as the infant exhibited a degree of suspicion and was reluctant to taste it when presented by the experimenter. Additionally, the mother was instructed to imitate the act of drinking from a bottle containing water. During the 45-second presentation of the bottle, in the event of a rejection, the experimenter offered the bottle three additional times before continuing with the experimental procedure (7).

Each bottle (TEX baby, Carrefour, Argentina) contained 50 mL of solution. The bottles were weighed before and after ingestion to the nearest 0.1 g. For each bottle, the experimenter, who was blind to the tastant, evaluated the infant’s liking of the tastant through the infant’s global behaviour by using a 5-point scale with the following specifications: 1 = “strong rejection”; 2 = “slight rejection”; 3 = “neutral reaction”; 4 = “slight acceptance”; and 5 = “strong acceptance” (8). The instruction for the experimenters was to consider the infant’s global behaviour, including his/her facial expressions. For example, if the infant spat out, frowned, pushed it away, and ceased drinking, the experimenter recorded a score of 1, indicating a “strong rejection”. In contrast, if the infant smiled, displayed a relaxed face, grabbed the bottle, and drank, the experimenter recorded a score of 5, indicating a “strong acceptance”(8).

*Definition of test variables*. The analysis was restricted to the infants who completed the taste acceptance test, consuming at least 1.0 g from at least 1 bottle of water and 1 bottle of sweet or fat tastant. For each tastant, an IR, which was based on the consumed volumes, and a LR, which was based on the experimenters’ judgment of the infant’s liking, were calculated and represented the acceptance of the tastant relative to water. For each tastant, the IR was defined as the ingestion volume of this tastant relative to the sum of ingestion volumes of this tastant and of water [i.e. total ingested volume of sweet solution/(total ingested volume of sweet solution + total ingested volume of water)]. Using a proportional variable makes comparisons across individuals possible, eliminating individual-related differences in sucking behaviour. Likewise, an LR was calculated for the scores of infant liking evaluated by the experimenter. By definition, each ratio may vary between 0 and 1; a ratio of 0.5 indicates indifference to the tastant relative to water. A ratio > 0.5 indicates preference for the tastant relative to water. A ratio < 0.5 indicates rejection of the tastant relative to water (7, 8).

2. *Twenty-four-hour Dietary Recall and Classification of Hyperpalatable Foods.* A 24-hour dietary recall of the infant’s intake on the day prior to the second visit was conducted (10). The recall was administered by a trained professional, who asked the mother to describe in detail every food item the infant had consumed the previous day. The energy and nutrient composition of each consumed food—kilocalories (kcal), carbohydrates, simple sugars, and fat—was calculated using data from the Food Analysis and Recording System (SARA, by its Spanish acronym *Sistema de Análisis y Registro de Alimentos*), developed by the Ministry of Health of Argentina (11). Based on the hyperpalatable food (HPF) classification criteria proposed by Fazzino et al (12), the foods consumed were categorized into: fat and sodium combination (F-SOD) when >40% of kcal came from fat and sodium content was ≥0.20% by weight; carbohydrates and sodium combination (C-SOD) when >40% of kcal came from carbohydrates and sodium content was ≥0.20% by weight; and fat and simple sugars combination (FS), when >20% of kcal came from fat and >20% of kcal from simple sugars. The number of foods consumed in each category was counted, and the total number of hyperpalatable foods consumed by each infant was summed and grouped under each HPF category and expressed as percentage of total food items consumed.

*3. Statistical methods.* Power calculations were not performed as the data were derived from a larger clinical trial (1). Statistical analyses were performed using the R statistical software version 4.3.0. Shapiro-Wilk test was applied to test normality. Continuous variables with normal distribution are presented as mean (standard deviation [SD]); non-normal variables are reported as median (Q1, Q3), and categorical data are summarized as frequency counts and percentages. Variables presenting a log-normal distribution were log-transformed for subsequent analyses and expressed as geometric mean (95% confidence interval (CI)). Multiple linear regression analysis was used to test the relationship of IRs, LRs or HPF categories with milk, infant plasma hormones concentrations, adjusting for potential confounders (maternal age, pre-pregnancy maternal body mass index, parity (primipara vs. multipara), adequacy of gestational weight gain, and infant BMI z-score at the moment of visit) when needed. These variables were identified a priori based on prior evidence from our group (1) and from other studies in the field. Regression coefficients were reported with their corresponding 95% CI. Effect size was reported as the incremental Cohen’s f² for the specific hormone in the linear regression models, with values of 0.02, 0.15, and 0.35 considered small, medium, and large effects, respectively (13). The correlation between infant BMI z-score at the time of the visit and IRs or LRs was assessed, but no significant associations were observed for any of the outcome variables. Finally, mediation analysis was conducted to examine whether the potential relationship between milk plasma hormone concentrations (exposure variables) and IR or LRs (outcome variables) was mediated by infant plasma hormone levels. The mediation analysis followed these steps: (1) it was tested whether the exposure variable was significantly associated with the outcome variable (total effect = Path *c*); (2) it was tested whether the exposure variable was significantly associated with the proposed mediator (Path *a*); (3) it was examined whether the mediator was significantly associated with the outcome variable (Path *b*); and (4) the significance of the indirect or mediation effect (the product of Path *a* and Path *b*) was tested (14). The direct effect (Path *c*') was defined as the impact of the exposure variable on the outcome variable, after adjusting for the mediator (without accounting for the influence of the mediator). The total effect was the sum of the direct and indirect effects **(Supplemental Figure 1)** (15, 16). Linear regression models were used for each step, and the indirect effect was tested for significance using the nonparametric Bootstrap confidence intervals with the Percentile Method, with a 95% CI (17), performed using the *Mediation* package in R. A **post-hoc directed acyclic graph (DAG)** was generated to illustrate the pattern of significant associations observed between milk and infant hormones and subsequent infant taste acceptance and HPF intake. The graph was produced using the *dagitty* package for graph specification and the *ggdag* package for visualization. All statistical tests were two-tailed and the significance level was set at p < 0.05. Given the exploratory nature of this study, adjustments for multiple testing (e.g., Bonferroni correction) were not applied.

**Supplemental Figure 1** Conceptual diagram of the investigated mediation analyses.

Path *a* indicates the path from maternal plasma or milk hormones concentration (exposure) to milk or infant plasma hormone concentrations or hyperpalatable foods (HPF) type (mediator). Path *b* indicates the path from milk or infant plasma hormone concentrations or HPF type (mediator) to IR or LR sweet or fat taste (outcome). Path *c* (Total effect) indicates the path from maternal plasma or milk hormones concentration (exposure) to IR or LR sweet or fat taste (outcome). The indirect effect can be estimated by multiplying Path *a* and Path *b*. Direct effect indicates the path from maternal plasma or milk hormones concentration (exposure) to IR or LR sweet or fat taste (outcome) when controlled for milk or infant plasma hormone concentrations or HPF type (mediator), this can be estimated as [Path c – (Path a*Path b)]. The model was adjusted for maternal age, pre-pregnancy maternal body mass index, parity (primipara vs. multipara), adequacy of gestational weight gain and z-score BMI at the moment of the test.

**Supplemental Figure 2:** Flowchart of participants' recruitment

**Supplemental Table 1:** Associations of infant sweet and fat taste acceptance (IR and LR) with milk insulin, cortisol, leptin, and LEAP2 concentrations.

|  | Unadjusted analysis | |  | | Adjusted analysis* | | | | Adjusted analysis** | | | | | | |  |
| --- | --- | --- | --- | --- | --- | --- | --- | --- | --- | --- | --- | --- | --- | --- | --- | --- |
|  | Beta (95% CI) | p | Cohen's f2 | Beta (95% CI) | | | p | | Cohen's f2 | | Beta (95% CI) | | p | | Cohen's f2 |  |
| ***Sweet Taste*** | | | | | | | | | | | | | | | |  |
| *Milk LEAP2 (ng/ml, log)* |  |  |  |  | |  | |  | |  | |  | |  | | |
| Ingestion Ratio (IR) | -0.02 (-0.35, 0.31) | 0.915 | 0.00 | -0.11 (-0.39, 0.18) | | 0.418 | | 0.59 | | -0.11 (-0.34, 0.11) | | 0.287 | | 1.84 | | |
| Liking Ratio (LR) | -0.01 (-0.16, 0.14) | 0.854 | 0.00 | -0.04 (-0.24, 0.15) | | 0.629 | | 0.27 | | -0.04 (-0.25, 0.17) | | 0.676 | | 0.05 | | |
| *Milk Insulin (μU/ml, log)* |  |  |  |  | |  | |  | |  | |  | |  | | |
| Ingestion Ratio (IR) | -0.03 (-0.21, 0.16) | 0.771 | 0.00 | -0.18 (-0.43, 0.06) | | 0.129 | | 0.16 | | -0.36 (-0.57, -0.14) | | **0.003** | | 0.84 | | |
| Liking Ratio (LR) | 0.01 (-0.12, 0.15) | 0.857 | 0.00 | -0.17 (-0.33, 0.00) | | **0.049** | | 0.31 | | -0.21 (-0.39, -0.04) | | **0.020** | | 0.49 | | |
| *Milk Leptin (ng/ml, log)* |  |  |  |  | |  | |  | |  | |  | |  | | |
| Ingestion Ratio (IR) | 0.17 (0.07; 0.27) | **0.003** | 0.59 | 0.16 (0.04, 0.28) | | **0.011** | | 0.51 | | 0.16 (0.04, 0.27) | | **0.013** | | 0.51 | | |
| Liking Ratio (LR) | 0.07 (-0.05, 0.18) | 0.252 | 0.07 | 0.13 (0.00, 0.25) | | **0.045** | | 0.30 | | 0.14 (0.03, 0.26) | | **0.018** | | 0.47 | | |
| *Milk Cortisol (ug/dl, log)* |  |  |  |  | |  | |  | |  | |  | |  | | |
| Ingestion Ratio (IR) | 0.10 (-0.03, 0.23) | 0.108 | 0.15 | 0.13 (-0.01, 0.26) | | 0.069 | | 0.26 | | 0.19 (0.04, 0.33) | | **0.014** | | 0.56 | | |
| Liking Ratio (LR) | 0.04 (-0.06, 0.13) | 0.426 | 0.03 | 0.07 (-0.04, 0.18) | | 0.171 | | 0.13 | | 0.06 (-0.05, 0.18) | | 0.274 | | 0.09 | | |
| ***Fat Taste*** |  |  |  |  | |  | |  | |  | |  | |  | | |
| *Milk LEAP2 (ng/ml, log)* |  |  |  |  | |  | |  | |  | |  | |  | | |
| Ingestion Ratio (IR) | 0.20 (-0.12, 0.42) | 0.204 | 0.14 | 0.28 (-0.04, 0.60) | | 0.077 | | 0.69 | | 0.29 (0.02, 0.56) | | **0.037** | | 1.22 | | |
| Liking Ratio (LR) | 0.09 (-0.27, 0.46) | 0.594 | 0.02 | 0.07 (-0.36, 0.50) | | 0.721 | | 0.44 | | -0.15 (-0.53, 0.22) | | 0.385 | | 1.37 | | |
| *Milk Insulin (μU/ml, log)* |  |  |  |  | |  | |  | |  | |  | |  | | |
| Ingestion Ratio (IR) | 0.04 (-0.10, 0.18) | 0.527 | 0.02 | 0.19 (0.00, 0.37) | | **0.047** | | 0.37 | | 0.21 (0.02, 0.39) | | **0.032** | | 0.49 | | |
| Liking Ratio (LR) | -0.12 (-0.21, -0.03) | **0.011** | 0.44 | -0.14 (-0.25, -0.04) | | **0.013** | | 0.58 | | -0.14 (-0.25, -0.03) | | **0.015** | | 0.61 | | |
| *Milk Leptin (ng/ml, log)* |  |  |  |  | |  | |  | |  | |  | |  | | |
| Ingestion Ratio (IR) | 0.01 (-0.14, 0.17) | 0.841 | 0.00 | 0.01 (-0.22, 0.24) | | 0.937 | | 0.00 | | 0.00 (-0.22, 0.23) | | 0.967 | | 0.00 | | |
| Liking Ratio (LR) | -0.07 (-0.17, 0.04) | 0.226 | 0.09 | -0.07 (-0.20, 0.06) | | 0.285 | | 0.10 | | -0.10 (-0.22, 0.03) | | 0.126 | | 0.22 | | |
| *Milk Cortisol (ug/dl, log)* |  |  |  |  | |  | |  | |  | |  | |  | | |
| Ingestion Ratio (IR) | 0.22 (0.07, 0.38) | **0.008** | 0.54 | 0.24 (0.03, 0.44) | | **0.026** | | 0.49 | | 0.22 (0.01, 0.44) | | **0.043** | | 0.43 | | |
| Liking Ratio (LR) | 0.06 (-0.06, 0.19) | 0.282 | 0.07 | 0.12 (-0.01, 0.25) | | 0.064 | | 0.32 | | 0.10 (-0.03, 0.23) | | 0.118 | | 0.22 | | |

Data are presented as the regression coefficient beta (95% confidence interval) from linear regression analyses. Values in bold indicate statistical significance. *Adjusted for maternal age, pre-pregnancy maternal body mass index, parity (primipara vs. multipara) and adequacy of gestational weight gain. **Adjusted for maternal age, pre-pregnancy maternal body mass index, parity (primipara vs. multipara), adequacy of gestational weight gain and z-score BMI at the moment of the test.

**Supplemental Table 2:** Associations of infant sweet and fat taste acceptance (IR and LR) with plasma hormone concentrations and anthropometric measurements.

|  | Unadjusted analysis | | | | Adjusted analysis* | | | | Adjusted analysis** | | | | | |
| --- | --- | --- | --- | --- | --- | --- | --- | --- | --- | --- | --- | --- | --- | --- |
|  | Beta (95% CI) | p | Cohen's f2 | Beta (95% CI) | | p | Cohen's f2 | Beta (95% CI) | | p | Cohen's f2 | |  |  |
| ***Sweet Taste*** | | | | | | | | | | | | | | |
| *LEAP2 (ng/ml, log)* |  |  |  |  | |  |  |  | |  | |  | |  |
| Ingestion Ratio (IR) | -0.02 (-0.42, 0.38) | 0.933 | 0.00 | 0.58 (0.16, 1.01) | | **0.012** | 1.02 | 0.64 (0.24, 1.04) | | **0.005** | | 1.54 | |  |
| Liking Ratio (LR) | -0.02 (-0.30, 0.25) | 0.861 | 0.00 | 0.29 (0.04, 0.55) | | **0.028** | 1.18 | 0.28 (0.05, 0.52) | | **0.023** | | 1.72 | |  |
| *Insulin (µU/ml, log)* |  |  |  |  | |  |  |  | |  | |  | |  |
| Ingestion Ratio (IR) | -0.10 (-0.21, 0.00) | **0.046** | 0.23 | -0.04 (-0.18, 0.09) | | 0.482 | 0.03 | -0.05 (-0.19, 0.10) | | 0.497 | | 0.03 | |  |
| Liking Ratio (LR) | -0.10 (-0.18, 0.02) | **0.022** | 0.33 | -0.08 (-0.17, 0.02) | | 0.110 | 0.19 | -0.07 (-0.18, 0.04) | | 0.190 | | 0.14 | |  |
| *Leptin (ng/ml, log)* |  |  |  |  | |  |  |  | |  | |  | |  |
| Ingestion Ratio (IR) | -0.16 (-0.24, -0.07) | **0.001** | 0.75 | -0.14 (-0.23, -0.05) | | **0.006** | 0.63 | -0.16 (-0.26, -0.06) | | **0.004** | | 0.77 | |  |
| Liking Ratio (LR) | -0.11 (-0.18, -0.05) | **0.002** | 0.71 | -0.11 (-0.18, -0.03) | | **0.008** | 0.57 | -0.12 (-0.21, -0.02) | | **0.021** | | 0.51 | |  |
| *Cortisol (ug/dl, log)* |  |  |  |  | |  |  |  | |  | |  | |  |
| Ingestion Ratio (IR) | -0.19 (-0.42, 0.02) | 0.104 | 0.15 | -0.34 (-0.53, -0.14) | | **0.002** | 1.08 | -0.34 (-0.55, -0.12) | | **0.004** | | 1.04 | |  |
| Liking Ratio (LR) | 0.01 (-0.16, 0.17) | 0.943 | 0.00 | 0.02 (-0.14, 0.18) | | 0.797 | 0.01 | 0.04 (-0.13, 0.21) | | 0.649 | | 0.03 | |  |
| *BMI z-score* |  |  |  |  | |  |  |  | |  | |  | |  |
| Ingestion Ratio (IR) | -0.08 (-0.20, 0.04) | 0.187 | 0.09 | -0.09 (-0.21, 0.04) | | 0.154 | 0.14 |  | |  | |  | |  |
| Liking Ratio (LR) | -0.07 (-0.13, -0.01) | **0.027** | 0.30 | -0.07 (-0.13, 0.00) | | **0.039** | 0.34 |  | |  | |  | |  |
| ***Fat Taste*** |  |  |  |  | |  |  |  | |  | |  | |  |
| *LEAP2 (ng/ml, log)* |  |  |  |  | |  |  |  | |  | |  | |  |
| Ingestion Ratio (IR) | -0.37 (-0.70, -0.03) | **0.033** | 0.37 | -0.47 (-0.83, -0.10) | | **0.017** | 0.63 | -0.46 (-0.82, -0.10) | | **0.018** | | 0.72 | |  |
| Liking Ratio (LR) | 0.30 (0.09, 0.50) | **0.008** | 0.69 | 0.32 (0.09, 0.56) | | **0.012** | 0.99 | 0.35 (0.07, 0.62) | | **0.018** | | 1.05 | |  |
| *Insulin (µU /ml, log)* |  |  |  |  | |  |  |  | |  | |  | |  |
| Ingestion Ratio (IR) | 0.04 (-0.11, 0.20) | 0.560 | 0.02 | 0.14 (-0.08, 0.36) | | 0.196 | 0.16 | 0.20 (0.00, 0.40) | | **0.048** | | 0.45 | |  |
| Liking Ratio (LR) | -0.02 (-0.12, 0.08) | 0.706 | 0.01 | -0.09 (-0.17, -0.01) | | **0.027** | 0.59 | -0.09 (-0.17, -0.01) | | **0.032** | | 0.62 | |  |
| *Leptin (ng/ml, log)* |  |  |  |  | |  |  |  | |  | |  | |  |
| Ingestion Ratio (IR) | -0.12 (-0.35, 0.10) | 0.266 | 0.08 | -0.37 (-0.61, -0.13) | | **0.006** | 0.84 | -0.42 (-0.57, -0.27) | | **<0.001** | | 3.01 | |  |
| Liking Ratio (LR) | 0.08 (-0.06, 0.22) | 0.252 | 0.08 | 0.20 (-0.01, 0.42) | | 0.064 | 0.32 | 0.18 (-0.08, 0.44) | | 0.158 | | 0.19 | |  |
| *Cortisol (ug/dl, log)* |  |  |  |  | |  |  |  | |  | |  | |  |
| Ingestion Ratio (IR) | 0.10 (-0.08, 0.28) | 0.259 | 0.09 | -0.03 (-0.26, 0.21) | | 0.816 | 0.11 | -0.02 (-0.28, 0.24) | | 0.861 | | 0.08 | |  |
| Liking Ratio (LR) | -0.16 (-0.28, -0.05) | **0.010** | 0.54 | -0.19 (-0.31, -0.06) | | **0.009** | 0.83 | -0.20 (-0.35, -0.06) | | **0.009** | | 0.91 | |  |
| *BMI z-score* |  |  |  |  | |  |  |  | |  | |  | |  |
| Ingestion Ratio (IR) | -0.07 (-0.19, 0.05) | 0.231 | 0.09 | -0.10 (-0.23, 0.04) | | 0.140 | 0.19 |  | |  | |  | |  |
| Liking Ratio (LR) | -0.06 (-0.15, 0.02) | 0.133 | 0.15 | -0.05 (-0.14, 0.04) | | 0.225 | 0.13 |  | |  | |  | |  |

Data are presented as the regression coefficient beta (95% confidence interval) from linear regression analyses. Values in bold indicate statistical significance. *Adjusted for maternal age, pre-pregnancy maternal body mass index, parity (primipara vs. multipara) and adequacy of gestational weight gain. **Adjusted for maternal age, pre-pregnancy maternal body mass index, parity (primipara vs. multipara), adequacy of gestational weight gain and z-score BMI at the moment of the test.

| **Supplemental Table 3:** Associations of milk hormone concentration with hyperpalatable foods intake. | | | | | | | | | | | | |
| --- | --- | --- | --- | --- | --- | --- | --- | --- | --- | --- | --- | --- |
|  | Unadjusted analysis | | | | Adjusted analysis* | | | Adjusted analysis** | | | | |
|  | Beta (95% CI) | p | Cohen's f2 | Beta (95% CI) | | p | Cohen's f2 | Beta (95% CI) | p | Cohen's f2 | |  |
| **% Total HPF** |  |  |  |  | |  |  |  |  |  | |  |
| Milk LEAP2 (ng/ml, log) | 0.00 (-0.01, 0.01) | 0.934 | 0.00 | 0.00 (-0.01, 0.01) | | 0.799 | 0.13 | 0.00 (-0.01, 0.01) | 0.899 | 0.26 | |  |
| Milk Insulin (μU/ml, log) | -3.91 (-10.70, 2.88) | 0.247 | 0.06 | -2.41 (-10.14, 5.32) | | 0.523 | 0.06 | -2.58 (-10.55, 6.00) | 0.572 | 0.06 | |  |
| Milk Leptin (ng/ml, log) | -6.35 (-12.09, -0.61) | **0.032** | 0.21 | -8.80 (-14.20, -3.40) | | **0.003** | 0.55 | -9.15 (-14.05, -4.25) | **0.001** | 0.76 | |  |
| Milk Cortisol (ug/dl, log) | 0.19 (-6.43, 6.82) | 0.952 | 0.00 | -0.50 (-7.53, 6.53) | | 0.883 | 0.02 | -0.48 (-7.71, 6.76) | 0.892 | 0.03 | |  |
| **% F-SOD** |  |  |  |  | |  |  |  |  | |  |  |
| Milk LEAP2 (ng/ml, log) | -5.82 (-13.35, 1.70) | 0.121 | 0.15 | -0.97 (-10.39, 8.44) | | 0.828 | 0.65 | -0.56 (-10.71, 9.60) | 0.907 | 0.59 | |  |
| Milk Insulin (μU/ml, log) | -0.12 (-4.40, 4.15) | 0.953 | 0.00 | -4.21 (-8.44, 0.02) | | 0.051 | 0.25 | -4.53 (-8.88, -0.18) | **0.042** | 0.27 | |  |
| Milk Leptin (ng/ml, log) | -0.48 (-4.08, 3.12) | 0.785 | 0.00 | 1.59 (-3.35, 6.53) | | 0.511 | 0.02 | 1.34 (-3.80, 6.48) | 0.593 | 0.01 | |  |
| Milk Cortisol (ug/dl, log) | 2.12 (-2.96, 7.20) | 0.397 | 0.03 | 0.24 (-4.78, 5.27) | | 0.920 | 0.05 | 0.39 (-4.67, 5.45) | 0.874 | 0.03 | |  |
| **% C-SOD** |  |  |  |  | |  |  |  |  |  | |  |
| Milk LEAP2 (ng/ml, log) | 2.70 (-9.70, 15.10) | 0.6516 | 0.01 | 11.38 (2.55, 20.21) | | **0.016** | 0.38 | 10.85 (0.76, 20.93) | **0.037** | 0.39 | |  |
| Milk Insulin (μU/ml, log) | -0.68 (-4.62, 3.26) | 0.726 | 0.01 | -1.37 (-4.56, 1.82) | | 0.382 | 0.08 | -1.43 (-4.76, 1.91) | 0.382 | 0.07 | |  |
| Milk Leptin (ng/ml, log) | 0.24 (-2.66, 3.14) | 0.864 | 0.00 | 0.11 (-2.71, 2.93) | | 0.936 | 0.00 | 0.02 (-2.92, 2.96) | 0.990 | 0.00 | |  |
| Milk Cortisol (ug/dl, log) | 0.39 (-3.11, 3.88) | 0.822 | 0.00 | -0.43 (-3.02, 2.16) | | 0.734 | 0.04 | -0.43 (-3.10, 2.24) | 0.739 | 0.03 | |  |
| **% FS** |  |  |  |  | |  |  |  |  |  | |  |
| Milk LEAP2 (ng/ml, log) | 3.38 (-7.97, 14.73) | 0.539 | 0.02 | 9.30 (-4.66, 23.26) | | 0.175 | 0.11 | 15.52 (1.82, 29.22) | **0.029** | 0.53 | |  |
| Milk Insulin (μU/ml, log) | 0.12 (-2.90, 3.14) | 0.932 | 0.00 | 0.99 (-3.06, 5.05) | | 0.614 | 0.02 | 0.50 (-3.59, 4.59) | 0.801 | 0.02 | |  |
| Milk Leptin (ng/ml, log) | -2.88 (-5.89, 0.13) | 0.060 | 0.16 | -1.81 (-5.20, 1.58) | | 0.280 | 0.06 | -1.79 (-5.21, 1.64) | 0.289 | 0.06 | |  |
| Milk Cortisol (ug/dl, log) | -0.60 (-4.88, 3.68) | 0.775 | 0.00 | -1.90 (-5.91, 2.11) | | 0.334 | 0.05 | -2.22 (-6.06, 1.62) | 0.242 | 0.10 | |  |

Data are presented as the regression coefficient beta (95% confidence interval) from linear regression analyses. Values in bold indicate statistical significance. *Adjusted for maternal age, pre-pregnancy maternal body mass index, parity (primipara vs. multipara) and adequacy of gestational weight gain. **Adjusted for maternal age, pre-pregnancy maternal body mass index, parity (primipara vs. multipara), adequacy of gestational weight gain and z-score BMI at the moment of the test. Abbreviations: HPF: hyper-palatable foods; F-SOD: fat and sodium; C-SOD: carbohydrates and sodium; FS, fat and simple sugars

| **Supplemental Table 4:** Associations of HPF intake with infant hormone concentrations and anthropometric measurements. | | | | | | | | | | |
| --- | --- | --- | --- | --- | --- | --- | --- | --- | --- | --- |
|  | Unadjusted analysis | | | Adjusted analysis* | | | Adjusted analysis** | | | |
|  | Beta (95% CI) | p | Cohen's f2 | Beta (95% CI) | p | Cohen's f2 | Beta (95% CI) | p | Cohen's f2 | |
| **% Total HPF** |  |  |  |  |  |  |  |  |  | |
| LEAP2 (ng/ml, log) | -14.21 (-33.71, 5.28) | 0.144 | 0.12 | -32.64 (-51.44, -13.85) | **0.002** | 0.79 | -32.08 (-50.92, -13.25) | **0.002** | 1.11 | |
| Insulin (µU /ml, log) | 3.59 ( -3.25, 10.44) | 0.290 | 0.05 | 1.47 (-6.76, 9.69) | 0.715 | 0.01 | 3.10 (-5.81, 12.00) | 0.477 | 0.03 | |
| Leptin (ng/ml, log) | 0.34 (-8.40, 9.08) | 0.937 | 0.00 | -1.65 (-11.27, 7.97) | 0.725 | 0.01 | -0.97 (-11.30, 9.35) | 0.846 | 0.00 | |
| Cortisol (ug/dl, log) | 12.45 (2.34, 22.56) | **0.018** | 0.28 | 12.57 (2.30, 22.83) | **0.019** | 0.35 | 14.63 (4.78, 24.48) | **0.006** | 0.55 | |
| BMI z-score | -2.37 (-7.55, 2.02) | 0.310 | 0.04 | -1.02 (-5.81 3.76) | 0.661 | 0.01 |  |  |  | |
| **% F-SOD** |  |  |  |  |  |  |  |  | |  |
| LEAP2 (ng/ml, log) | -12.28 (-23.34, -1.23) | **0.031** | 0.30 | -12.93 (-22.94, -2.92) | **0.015** | 1.50 | -13.32 (-24.00, -2.64) | **0.018** | 1.44 | |
| Insulin (µU /ml, log) | -0.20 (-5.19, 4.79) | 0.935 | 0.00 | 1.34 (-4.59, 7.28) | 0.643 | 0.01 | 3.18 (-3.01, 9.37) | 0.297 | 0.06 | |
| Leptin (ng/ml, log) | -2.21 (-8.73, 4.30) | 0.490 | 0.02 | -1.66 (-9.26, 5.94) | 0.654 | 0.01 | -0.98 (-9.12, 7.15) | 0.803 | 0.00 | |
| Cortisol (ug/dl, log) | -0.09(-8.65, 8.48) | 0.984 | 0.00 | -1.01(-9.71, 7.68) | 0.810 | 0.00 | 0.16 (-9.85, 10.17) | 0.974 | 0.01 | |
| BMI z-score | -2.19 (-5.39 1.02) | 0.173 | 0.08 | -1.55 (-4.55 1.46) | 0.297 | 0.05 |  |  |  | |
| **% C-SOD** |  |  |  |  |  |  |  |  |  | |
| LEAP2 (ng/ml, log) | -9.524 (-21.66, 2.62) | 0.117 | 0.14 | -13.67 (-24.63, -2.72) | **0.018** | 0.46 | -13.59 (-24.90, -2.27) | **0.022** | 0.48 | |
| Insulin (µU /ml, log) | 0.73 (-2.25, 3.71) | 0.617 | 0.01 | -0.70 (-3.28, 1.89) | 0.581 | 0.01 | -1.51 (-4.50, 1.48) | 0.305 | 0.06 | |
| Leptin (ng/ml, log) | -0.82 (-5.59, 3.95) | 0.726 | 0.01 | -1.87 (-6.62, 2.87) | 0.422 | 0.03 | -2.23 (-7.32, 2.86) | 0.371 | 0.04 | |
| Cortisol (ug/dl, log) | 3.59 (-2.24, 9.42) | 0.215 | 0.07 | 3.54 (-0.88, 7.96) | 0.110 | 0.15 | 3.69 (-1.12, 8.50) | 0.124 | 0.15 | |
| BMI z-score | -0.83 (-3.40 1.75) | 0.513 | 0.02 | 0.07 (-1.85 1.99) | 0.939 | 0.00 |  |  |  | |
| **% FS** |  |  |  |  |  |  |  |  |  | |
| LEAP2 (ng/ml, log) | 10.85 (-1.67, 23.37) | 0.085 | 0.18 | 7.49 (-4.97, 19.94) | 0.218 | 0.41 | 9.16 (-5.23, 23.56) | 0.192 | 0.41 | |
| Insulin (µU /ml, log) | 2.33 (-1.25, 5.91) | 0.192 | 0.07 | 0.31 (-3.69, 4.33) | 0.870 | 0.00 | 0.43 (-4.02, 4.88) | 0.843 | 0.00 | |
| Leptin (ng/ml, log) | 2.36 (-3.37, 8.09) | 0.404 | 0.03 | 0.83 (-4.93, 6.60) | 0.766 | 0.00 | 1.23 (-4.68, 7.13) | 0.670 | 0.01 | |
| Cortisol (ug/dl, log) | 5.16 (-0.59, 10.92) | 0.077 | 0.14 | 5.68 (0.02, 11.35) | **0.049** | 0.21 | 6.36 (0.36, 12.35) | **0.039** | 0.25 | |
| BMI z-score | -0.96 (-3.87 1.95) | 0.502 | 0.02 | -1.94 (-4.60 0.71) | 0.142 | 0.12 |  |  |  | |
|  |  |  |  |  |  |  |  |  |  | |
|  |  |  |  |  |  |  |  |  |  | |

Data are presented as the regression coefficient beta (95% confidence interval) from linear regression analyses. Values in bold indicate statistical significance. *Adjusted for maternal age, pre-pregnancy maternal body mass index, parity (primipara vs. multipara) and adequacy of gestational weight gain. **Adjusted for maternal age, pre-pregnancy maternal body mass index, parity (primipara vs. multipara), adequacy of gestational weight gain and z-score BMI at the moment of the test. Abbreviations: HPF: hyper-palatable foods; F-SOD: fat and sodium; C-SOD: carbohydrates and sodium; FS, fat and simple sugars

References

1.	Kruger AL, Malpeli A, Sala M, Casado C, Mendez I, Fotia L, et al. The Concentration of Liver-Expressed Antimicrobial Peptide 2 in Human Milk and Infant Plasma Is Positively Associated with Adiposity and Body Weight in the First Year of Life. The Journal of nutrition. 2024;154(11):3388-99. doi: 10.1016/j.tjnut.2024.09.008.

2.	Labbok MH, Belsey M, Coffin CJ. A call for consistency in defining breast-feeding. American journal of public health. 1997;87(6):1060-1. doi: 10.2105/ajph.87.6.1060.

3.	Edtion ed. In: Rasmussen KM, Yaktine AL, eds. Weight Gain During Pregnancy: Reexamining the Guidelines. Washington (DC), 2009.

4.	Kruger AL, Malpeli A, Sala M, Casado C, Mendez I, Fotia L, Tournier A, Fasano MV, Andreoli MF. Human milk cortisol is inversely associated with infant BMI and mediates the association between maternal plasma and infant salivary cortisol concentrations. Int J Obes (Lond). 2025;49(8):1632-41. doi: 10.1038/s41366-025-01815-4.

5.	Use WHOECoPSt, Interpretation of A, World Health O. Physical status : the use of and interpretation of anthropometry , report of a WHO expert committee. Geneva: World Health Organization, 1995.

6.	WHO Multicentre Growth Reference Study Group. WHO Child Growth Standards: Head circumference-for-age, arm circumference-for-age, triceps skinfold-for-age and subscapular skinfold-for-age: Methods and development. Geneva: World Health Organization, 2007.

7.	Schwartz C, Chabanet C, Szleper E, Feyen V, Issanchou S, Nicklaus S. Infant Acceptance of Primary Tastes and Fat Emulsion: Developmental Changes and Links with Maternal and Infant Characteristics. Chemical senses. 2017;42(7):593-603. doi: 10.1093/chemse/bjx040.

8.	Schwartz C, Issanchou S, Nicklaus S. Developmental changes in the acceptance of the five basic tastes in the first year of life. The British journal of nutrition. 2009;102(9):1375-85. doi: 10.1017/S0007114509990286.

9.	Beauchamp GK, Moran M. Dietary experience and sweet taste preference in human infants. Appetite. 1982;3(2):139-52. doi: 10.1016/s0195-6663(82)80007-x.

10.	Beaton E, Wright J, Devenish G, Do L, Scott J. Relative Validity of a 24-h Recall in Assessing Intake of Key Nutrients in a Cohort of Australian Toddlers. Nutrients. 2018;10(1). doi: 10.3390/nu10010080.

11.	Ministerio de Salud de la Nación. SARA 2 : tabla de composición química de alimentos para Argentina : compilación para ENNyS 2. Ciudad Autónoma de Buenos Aires 2022.

12.	Fazzino TL, Rohde K, Sullivan DK. Hyper-Palatable Foods: Development of a Quantitative Definition and Application to the US Food System Database. Obesity. 2019;27(11):1761-8. doi: 10.1002/oby.22639.

13.	Cohen J. Statistical power analysis for the behavioral sciences: Routledge, 2013.

14.	Baron RM, Kenny DA. The moderator-mediator variable distinction in social psychological research: conceptual, strategic, and statistical considerations. Journal of personality and social psychology. 1986;51(6):1173-82. doi: 10.1037//0022-3514.51.6.1173.

15.	Christensen SH, Lewis JI, Larnkjaer A, Frokiaer H, Allen LH, Molgaard C, Michaelsen KF. Associations between maternal adiposity and appetite-regulating hormones in human milk are mediated through maternal circulating concentrations and might affect infant outcomes. Frontiers in nutrition. 2022;9:1025439. doi: 10.3389/fnut.2022.1025439.

16.	MacKinnon DP, Fairchild AJ, Fritz MS. Mediation analysis. Annual review of psychology. 2007;58:593-614. doi: 10.1146/annurev.psych.58.110405.085542.

17.	Mackinnon DP, Lockwood CM, Williams J. Confidence Limits for the Indirect Effect: Distribution of the Product and Resampling Methods. Multivariate behavioral research. 2004;39(1):99. doi: 10.1207/s15327906mbr3901_4.
